# Adherence to colonoscopy recommendations for first-degree relatives of young patients diagnosed with colorectal cancer

**DOI:** 10.6061/clinics/2015(10)07

**Published:** 2015-10

**Authors:** Guilherme H Garcia, Rachel P Riechelmann, Paulo M Hoff

**Affiliations:** I Faculdade de Medicina da Universidade de São Paulo, São Paulo, SP, Brazil; II Instituto do Cancer do Estado de São Paulo; Disciplina de Radiologia e Oncologia, São Paulo, SP, Brazil

**Keywords:** Colorectal Cancer Screening, Colorectal Cancer, Colonoscopy, American College of Gastroenterology Guidelines, Hereditary Non-Polyposis Colorectal Cancer, Lynch Syndrome, Adenomatous Familiar Polyposis

## Abstract

**OBJECTIVES:**

Colorectal cancer is the third leading cause of cancer death in the United States. The American College of Gastroenterology recommends screening for first-degree relatives of patients diagnosed with colorectal cancer before the age of 50. A colonoscopy is one of the most commonly recommended exams due to its specificity and the possibility to resect pre-malignant lesions. Nevertheless, the rate of physician adherence to this recommendation is unknown.

**METHODS:**

This transversal study was performed at a major cancer center in Brazil with 62 patients, aged 18 to 50, who completed a questionnaire on information received from their physicians regarding screening their first-degree relatives. We used the answers from patients who provided explicit consent.

**RESULTS:**

Two hundred and three patients were eligible to participate and 93 (45.8%) agreed to complete the questionnaire. Twenty-three questionnaires (24.73%) were returned and 39 were completed by telephone. Of the patients who answered the questionnaire, 39 (62.9%) had received a colonoscopy recommendation for their first-degree relatives and 23 (37.1%) were not informed of the recommendation. Among the patients who received the recommendations, 20.51% affirmed that all relatives completed the exam and 51.28% stated that no relatives completed the exam.

**DISCUSSION:**

The adherence rate of our physicians to the ACG guideline recommendations was 62.9%. Considering that our study was performed at a leading center for cancer treatment in Latin America, we had expected better adherence. The results show that adherence to the colorectal cancer screening recommendations for high-risk patients must be improved.

## INTRODUCTION

Colorectal cancer is the third most common malignant disease and the third most frequent cause of cancer-related death in the United States 1. Despite improved results from chemotherapy and biological agents over recent years, an early diagnosis remains an important factor in determining a better prognosis. The chance of developing colorectal cancer is influenced by lifestyle, age and a familial history of colorectal cancer 3. Familial history is the most well studied of the risk factors. Many hereditary syndromes associated with colorectal cancer have been described, such as adenomatous familiar polyposis (FAP) and hereditary non-polyposis colorectal cancer. To enhance early diagnoses, especially in patients with a familial history of colorectal cancer, certain institutions, such as the American College of Gastroenterology, the American Cancer Society, the US Multi-Society Task Force on Colorectal Cancer and the American College of Radiology have developed guidelines for clinical practice regarding colorectal cancer screening 8,9.

Evidence from different studies has indicated reduced mortality in sporadic colorectal cancer cases due to screening using different methods 11-16 and colonoscopy is one of the most effective screening exams (8-16,17,2,7,18-33).

Despite the importance of screening programs in colorectal cancer prevention and mortality reduction, we know little regarding physician adherence to the guideline recommendations. Based on the recognized impact of colorectal cancer screening, which reduces mortality, we performed this study to evaluate physician adherence to the American College of Gastroenterology's recommendations on colonoscopy for patients' relatives at high risk of colorectal cancer.

## METHODS

This was a transversal study that evaluated physician adherence to screening recommendations for the first-degree relatives of patients who were diagnosed with colorectal cancer before the age of 50 at a major cancer center (Instituto do Câncer do Estado de São Paulo do Hospital das Clínicas da Faculdade de Medicina da Universidade de São Paulo, Brazil).

The study was approved by the institutional ethics committee.

We provided questionnaires to patients who attended this tertiary center between July 2007 and January 2010. The questionnaire evaluated the information that patients received from their physicians on screening options for their first-degree relatives. Eligible patients were contacted by phone and invited to participate in the study. Through this call, the patient had an opportunity to ask questions related to the study. The patients who accepted the opportunity to participate in the study received the questionnaire by mail.

The patients were instructed to complete the questionnaire and informed consent forms and return them to the hospital. Incomplete questionnaires were completed by telephone. The patients were contacted by telephone when the questionnaire were not sent back in two months.

In addition to the information provided by the questionnaire, other specific information, such as the date of diagnosis, treatments received, cancer histological type, tumor localization and cancer stage, were obtained from patient records after we received patient consent.

The data obtained from the questionnaires and through the patient records were tabulated. The results are shown below.

### Patient selection

All patients diagnosed with colorectal cancer before the age of 50 were identified based on an administrative list. Patients with International Classification of Diseases 18, 19 and 20 (colon, rectosigmoid junction and rectum) were used. Patients must have fulfilled the inclusion criteria to be eligible for the study.

### Eligibility

Patients were between 18 and 50 years of age and had been diagnosed with colorectal cancer and completed all treatments or were in treatment with more than 12 months of follow-up.

Patients with neuropsychiatric diseases that prevented consent or understanding and answering the questionnaire were excluded.

### Questionnaire description

The questionnaire contained demographic data, data regarding the patient's disease and questions regarding the recommendations that the patients received from their physicians.

Additional demographic data and information regarding the disease were collected from patient records. The obtained information included the date of birth, gender, date of diagnosis, treatments performed, tumor stage, tumor location and histology.

The questionnaire evaluated whether the physicians recommended screening with a colonoscopy or another screening exam to the patients' first-degree relatives.

Except for colonoscopy, we did not specify another exam to avoid influencing patient answers.

Finally, the patients whose doctors recommended a screening exam for their relatives were asked about the content of this recommendation. We asked the patient the age that their siblings should begin screening and how often the exam should be completed.

### Data analysis

The main goal of this study was evaluate physician adherence to colonoscopy recommendations for patients with a high risk of developing colorectal cancer.

The literature data are limited for supporting a calculation of the sample size required for this study and we estimated a sample size of between 80 and 100 patients.

The questionnaire that the patients answered allowed us to generate categorical variables and provide a descriptive data analysis, wherein variables were described as percentages.

We compared the sample obtained in this study with the total population, which we considered all patients who met the criteria. A simple t test was used to compare age, and two Mann-Whitney tests were performed to compare tumor localization and cancer stage between the two groups

## RESULTS

We selected the first 400 patients less than 50 years of age who were diagnosed with colorectal cancer and admitted to the hospital between June 2007 and January 2010. Among these patients, we selected those that fulfilled the inclusion criteria. Patients with metastasis (tumor stage IV) were excluded from the study because they were considered to be in treatment and, therefore, ineligible. Among the 400 patients, 11 (2.75%) were deceased when the study began. The records of another 27 patients (6.75%) were incomplete without information on cancer stage and metastasis. Thus, these patients were excluded from the study. An additional 158 patients (39.5%) presented with metastasis or were under clinical investigation and were thus also excluded.

Thus, 204 patients (51%) were considered eligible for the study. One of the patients was 15 years of age and was excluded. The other 203 patients received up to four phone calls. In these phone calls, the patients were invited to participate in the research. Of these 203 patients, 93 (45.8%) agreed to participate, 46 patients (22.7%) could not be contacted and, finally, 64 patients (31.5%) refused to participate. The questionnaires and terms of informed consent were sent to the 93 patients who agreed to participate.

Of the 93 patients who received the questionnaire, 23 returned it for an initial adherence rate of 24.73% (23/93). To improve adherence, the researchers phoned the patients and obtained 39 additional responses, which yielded a total of 62 patients. During the research, of the 93 patients, 6 patients died and another two patients who were initially included in the study were not diagnosed with colorectal cancer and were seen at the hospital due to a familial history of FAP. Therefore, among the 93 patients who were initially considered eligible to answer the questionnaire, 85 were truly eligible.

We obtained answers from 62 of the 85 patients, which is an adherence rate of 72.94%.

Among the patients who did not answer the questionnaire, 9 changed their telephone number and were impossible to contact. Another 14 patients did not answer the phone calls.

The sample profile (62 patients) is shown in Table 1.

### Population study and sample

Two hundred and three patients were considered eligible to participate the study. Among these, 62 patients (30.54%) comprised the sample. A simple test t showed that the sample had the same median age of the total population (43.38 and 41.91 years, respectively). These two groups were compared using a Mann-Whitney non-parametric test for tumor location and cancer stage, and there were no significant differences between the groups (*p*=0.9671 and *p*=0.7799, respectively).

### Questionnaires analyses

The study evaluated physician adherence to colonoscopy recommendations for first-degree relatives of young patients diagnosed with colorectal cancer. Of the 62 patients in the research, 39 (62.9%) received a colonoscopy recommendation for their first-degree relatives. The other 23 patients (37.1%) were not informed of the recommendation.

Among the 23 patients who had not received the colonoscopy recommendation, 2 received a fecal occult blood test (FOBT) recommendation. Twenty-one patients (33.88%) did not receive a screening exam recommendation for their first-degree relatives.

Of the 39 patients who received a colonoscopy recommendation for their first-degree relatives, 26 (66.7%) received information regarding the screening age, 9 (23%) did not remember whether they received the information and 4 (10.3%) did not receive the information from their physicians. One patient received incomplete information.

Information regarding the frequency of colonoscopy was provided to 20 patients (51.28%). Fifteen patients (38.46%) did not remember, and 4 patients (10.26%) never received the information. The recommended screening frequency was correct for all patients who received the recommendation.

The study also evaluated adherence of the patient and patient's relatives to the physician recommendations. Among the 39 patients who received the information, 8 patients (20.51%) affirmed that all of their siblings underwent a colonoscopy, 10 patients (25.64%) stated that some siblings completed the colonoscopy and, finally, 20 patients (51.28%) stated that no sibling was screened. One patient did not know if their siblings had been examined.

The patients whose siblings did not complete the colonoscopy were asked to provide one or more reasons that explain why their relatives did not complete the exam. The most common causes were fear, ignorance of the importance of completing the exam, difficulty accessing the exam in the public health system and disinterest of the relatives (Table 2).

## DISCUSSION

This transversal study evaluated 62 patients at a major cancer center in Brazil. The sample was similar to the total population of the study with regard to aspects such as age, tumor site and tumor stage. The rate of patients who received colorectal cancer screening recommendations for their first-degree relatives based on the American College of Gastroenterology guidelines was 62.9%. Among those patients, 20.51% affirmed that all relatives completed the exam and 51.28% indicated that no relative completed the exam.

The literature on colorectal cancer screening is vast and many studies have compared examination methods and reported new exam techniques or discussed a local experience with screening. However, data on screening for the general population are scarce and the authors found no data on how often physicians recommend screening to the first-degree relatives of patients diagnosed with colorectal cancer before the age of 50.

The publication we considered most relevant to this subject is a CDC report with results of a population survey using the Behavioral Risk Factor Surveillance System (BRFSS) (17). Through the BRFSS, a randomized population in the US at ≥50 years old was evaluated for use of the FOBT and/or sigmoidoscopy or colonoscopy. In 2006, 60.8% of the respondents reported having completed a FOBT one year before the survey and 55.7% reported completing a sigmoidoscopy or colonoscopy within the ten years before the survey. Finally, 29.5% of the respondents reported never being tested.

The BRFSS evaluated individuals in the general population and not necessarily those with a high risk for developing colorectal cancer; nevertheless, 60.8% of respondents had completed at least one type of screening exam. Our study showed that, in a hospital that specializes in cancer treatment, only 62.9% of patients diagnosed with colorectal cancer received information from their doctors recommending that their first-degree relatives complete a screening examination. It should be noted that the relatives of these patients were individuals with a high risk for developing colorectal cancer. Even more worrisome is the fact that, even when the patient received the appropriate recommendations, more than 50% of the families completely ignored them.

Studies in which patients receive questionnaires by mail and must return them to researchers are subject to a natural selection bias. This study was also subjected to an appraiser bias because many questionnaires were completed by telephone. Other methodological limitations of this study include a low number of patients and relatively low rate of patient adherence to the study. We also note that this is a single-center study conducted in a public academic hospital and may not reflect reality at other hospitals.

The hospital where this research took place is a leading center in cancer treatment in Latin America and, thus, we expected a higher adherence rate to ACG guidelines. The results are far from our ideal and showed that colorectal cancer screening for high-risk patients must improve. The best way to improve the rate of screening in high-risk patients is teaching and encouraging doctors to recommend a colonoscopy. The low adherence of the patientś relatives to the physicians' recommendations is another aspect that should improve. The patients and their relatives should be educated on the importance of screening for preventing and reducing of mortality in colorectal cancer and patient access to a colonoscopy should be facilitated.

This transversal study, despite its methodological limitations, takes an unprecedented approach and focuses on a neglected subject that has great importance for in public health: doctor use of guideline recommendations. This study takes a different approach and evaluates the rate of physician adherence rather than patient adherence; it also indicates a need for constant education and reinforcement of screening guideline recommendations for doctors.

## Figures and Tables

**Table 1:** Sample profile.

Age	Number	Percentage
18-20	0	0
21-30	3	4.83
31-40	14	22.58
41-50	45	72.59
Cancer stage		
I	4	6.45
IIA	15	24.2
IIB	8	12.9
IIIA	4	6.45
IIIB	14	22.58
IIIC	7	11.29
Data not available	10	16.13
Tumor location		
Colon (right, left and sigmoid)	36	58.06
Rectosigmoid junction	3	4.84
Rectal	23	37.1

**Table 2:** Questionnaire analysis.

	Total	Percentage
Did patients receive a colonoscopy recommendation for their first-degree relatives?		
Yes	39	62.9
No	23	37.1
Did patients receive a recommendation for other screening exams?		
Yes	2	8.69
No	21	91.31
Did patients receive adequate information about screening age?		
Yes	26	66.7
No	4	10.3
Do not remember	9	23
Adherence of patients and their relatives to the physiciańs recommendations		
All relatives received a colonoscopy	8	20.51
Some relatives received a colonoscopy	10	25.64
No relative received a colonoscopy	20	51.28
